# Supplementary material for: Effective Strategies for Managing COVID-19 Emergency Restrictions for Adults with Severe ASD in a Daycare Center in Italy
Source: Brain Sci. 2020 Jul 9;10(7):436. doi: 10.3390/brainsci10070436 (PMC7408508; doi:10.3390/brainsci10070436)
Supplement: Supplementary file 1 [file brainsci-10-00436-s001.docx]

Supplementary Materials: Effective Strategies for Managing COVID-19 Emergency Restrictions for Adults with Severe ASD in a Daycare Center in Italy

Natascia Brondino*, Stefano Damiani and Pierluigi Politi

**Table S1:** additional information about the sample.

| **ID** | **Sex** | **Age** | **NV** | **Leiter 3** | **ADI-R SO** | **ADI-R CO** | **ADI-R**  **RB** | **ADI-R DEV** | **ADOS** | **ABC TOT T0** | **ABC HYP T0** | **ABC IRR T0** | **ABC SWL T0** | **ABC STE T0** | **ABC INS T0** | **ABC TOT T1** | **ABC HYP T1** | **ABC IRR T1** | **ABC SWL T1** | **ABC STE T1** | **ABC INS T1** |
| --- | --- | --- | --- | --- | --- | --- | --- | --- | --- | --- | --- | --- | --- | --- | --- | --- | --- | --- | --- | --- | --- |
| 1 | M | 28 | X | - | 18 | 8 | 8 | 5 |  | 20 | 2 | 6 | 2 | 10 | 0 | 26 | 2 | 8 | 2 | 14 | 0 |
| 2 | M | 32 |  | 45 | 18 | 12 | 8 | 4 |  | 27 | 2 | 5 | 2 | 8 | 10 | 14 | 0 | 0 | 2 | 6 | 6 |
| 3 | M | 23 | X | - | 24 | 8 | 6 | 5 |  | 42 | 0 | 20 | 14 | 8 | 0 | 22 | 0 | 8 | 10 | 4 | 0 |
| 4 | F | 18 | X | - | 24 | 8 | 4 | 5 |  | 20 | 0 | 4 | 12 | 4 | 0 | 13 | 0 | 1 | 8 | 4 | 0 |
| 5 | M | 18 |  | 50 | 18 | 12 | 6 | 3 |  | 44 | 16 | 10 | 8 | 10 | 0 | 38 | 12 | 6 | 10 | 10 | 0 |
| 6 | M | 19 |  | 50 | 16 | 16 | 8 | 3 |  | 28 | 4 | 5 | 5 | 8 | 6 | 12 | 2 | 2 | 0 | 6 | 2 |
| 7 | M | 27 | X | - | 24 | 8 | 6 | 5 |  | 36 | 16 | 3 | 2 | 15 | 0 | 46 | 23 | 10 | 2 | 13 | 0 |
| 8 | M | 27 |  | 40 | 18 | 12 | 4 | 5 |  | 7 | 0 | 1 | 0 | 6 | 0 | 11 | 0 | 5 | 0 | 6 | 0 |
| 9 | M | 30 |  | 40 | 18 | 18 | 8 | 3 |  | 23 | 0 | 2 | 1 | 12 | 8 | 26 | 0 | 2 | 0 | 12 | 12 |
| 10 | F | 24 |  | - | 18 | 12 | 8 | 5 |  | 34 | 8 | 10 | 4 | 12 | 0 | 50 | 8 | 18 | 4 | 18 | 2 |
| 11 | F | 16 |  | 50 | 16 | 12 | 6 | 3 |  | 20 | 0 | 4 | 2 | 10 | 4 | 24 | 0 | 8 | 2 | 10 | 4 |
| 12 | M | 20 |  | - | 24 | 18 | 6 | 4 |  | 32 | 0 | 10 | 4 | 13 | 5 | 17 | 0 | 3 | 2 | 7 | 5 |
| 13 | M | 25 |  | 43 | 16 | 8 | 8 | 4 |  | 33 | 4 | 8 | 0 | 15 | 6 | 24 | 2 | 2 | 0 | 10 | 10 |
| 14 | F | 21 |  | 45 | 18 | 10 | 8 | 4 |  | 19 | 5 | 0 | 2 | 10 | 2 | 16 | 4 | 0 | 3 | 7 | 2 |
| 15 | M | 16 |  | 50 | 14 | 8 | 3 | 3 |  | 5 | 0 | 0 | 1 | 4 | 0 | 5 | 0 | 0 | 1 | 4 | 0 |
| 16 | M | 23 | X | - | 24 | 8 | 8 | 4 |  | 33 | 0 | 20 | 2 | 11 | 0 | 25 | 0 | 13 | 2 | 10 | 0 |
| 17 | M | 19 |  | - | 20 | 16 | 8 | 3 |  | 20 | 4 | 6 | 4 | 4 | 2 | 23 | 4 | 6 | 6 | 4 | 3 |
| 18 | F | 23 |  | 69 | 14 | 8 | 3 | 3 | Communication **5**  Social Interaction **9**  Total **14**  Imagination **1**  Stereotype **5** | 4 | 0 | 0 | 0 | 3 | 1 | 10 | 0 | 0 | 2 | 6 | 2 |

Legend: NV = non-verbal; ADI-R SO = reciprocal social interactions domain; ADI-R CO = language/communication domain; ADI-R RB = repetitive behaviors/interests domain; ADI-R DEV = abnormality of development evident at or before 36 months domain; ABC HYP = hyperactivity subscale; ABC IRR = irritability subscale; ABC SWL = social withdrawal/lethargy subscale; ABC STE = stereotype subscale; ABC INS = inappropriate speech subscale.

**Additional analyses**

Paired-sample t-test for ABC subscales: Hyperactivity (t = 0.44, *p* = 0.66); irritability (t = 0.99, *p* = 0.33); social withdrawal/lethargy (t = 1.04, *p* = 0.31); stereotype (t = 0.94, *p* = 0.36); inappropriate speech (t = -0.46, *p* = 0.65).
